# Blockade of ITGA2 Induces Apoptosis and Inhibits Cell Migration in Gastric Cancer

**DOI:** 10.1186/s12575-018-0073-x

**Published:** 2018-05-01

**Authors:** Yu-Chang Chuang, Hsin-Yi Wu, Yu-Ling Lin, Shey-Cherng Tzou, Cheng-Hsun Chuang, Ting-Yan Jian, Pin-Rong Chen, Yuan-Ching Chang, Chi-Hsin Lin, Tse-Hung Huang, Chao-Ching Wang, Yi-Lin Chan, Kuang-Wen Liao

**Affiliations:** 1 Departmet of Biological Science and Technology, National Chiao Tung University, 75 Bo-Ai Street, Hsinchu, 300 Taiwan, Republic of China; 2 Institute of Molecular Medicine and Bioengineering, National Chiao Tung University, 75 Bo-Ai Street, Hsinchu, 300 Taiwan, Republic of China; 3 Center for Bioinformatics Research, National Chiao Tung University, Hsinchu, Taiwan, Republic of China; 4 Department of Surgery, Mackay Memorial Hospital, Taipei, Taiwan, Republic of China; 5 Department of Medical Research, MacKay Memorial Hospital, Taipei, Taiwan, Republic of China; 6 Department of Traditional Chinese Medicine, Chang Gung Memorial Hospital, Keelung, Taiwan, Republic of China; 7 School of Traditional Chinese Medicine, Chang Gung University, Taoyuan, Taiwan, Republic of China; 8 School of Nursing, National Taipei University of Nursing and Health Sciences, Taipei, Taiwan, Republic of China; 9 Department of Life Science, Chinese Culture University, 55, Hwa-Kang Rd., Yang-Ming-Shan, Taipei, 11114 Taiwan, Republic of China; 10 College of Biological Science and Technology, National Chiao Tung University, Hsinchu, Taiwan, Republic of China; 11 Graduate Institute of Medicine, College of Medicine, Kaohsiung Medical University, Kaohsiung, Taiwan, Republic of China; 12 Department of Biotechnology and Bioindustry Sciences, National Cheng Kung University, Tainan, Taiwan, Republic of China

**Keywords:** Integrin alpha-2, ITGA2, Gastric cancer, Cell migration, Proliferation, Apoptosis, Therapeutic target

## Abstract

**Background:**

Gastric cancer is currently the fourth leading cause of cancer-related death worldwide. Gastric cancer is often diagnosed at advanced stages and the outcome of the treatment is often poor. Therefore, identifying new therapeutic targets for this cancer is urgently needed. Integrin alpha 2 (ITGA2) subunit and the beta 1 subunit form a heterodimer for a transmembrane receptor for extracellular matrix, is an important molecule involved in tumor cell proliferation, survival and migration. Integrin α2β1 is over-expressed on a variety of cancer cells, but is low or absent in most normal organs and resting endothelial cells.

**Results:**

In this report, we assessed the ITGA2 as the potential therapeutic target with the bioinformatics tools from the TCGA dataset in which composed of 375 gastric cancer tissues and 32 gastric normal tissues. According to the information from the Cancer Cell Line Encyclopedia (CCLE) database, the AGS cell line with ITGA2 high expression and the SUN-1 cell line with low expression were chosen for the further investigation. Interestingly, the anti-ITGA2 antibody (at 3 μg/ml) inhibited approximately 50% survival of the AGS cells (over-expressed ITGA2), but had no effect in SNU-1 cells (ITGA2 negative). The extents of antibody-mediated cancer inhibition positively correlated with the expression levels of the ITGA2. We further showed that the anti-ITGA2 antibody induced apoptosis by up-regulating the RhoA-p38 MAPK signaling to promote the expressions of Bim, Apaf-1 and Caspase-9, whereas the expressions of Ras and Bax/Bcl-2 were not affected. Moreover, blocking ITGA2 by the specific antibody at lower doses also inhibited cell migration of gastric cancer cells. Blockade of ITGA2 by a specific antibody down-regulated the expression of N-WASP, PAK and LIMK to impede actin organization and cell migration of gastric cancer cells.

**Conclusions:**

Here, we showed that the mRNA expression levels of ITGA2 comparing to normal tissues significantly increased. In addition, the results revealed that targeting integrin alpha 2 subunit by antibodies did not only inhibit cell migration, but also induce apoptosis effect on gastric cancer cells. Interestingly, higher expression level of ITGA2 led to significant effects on apoptosis progression during anti-ITGA2 antibody treatment, which indicated that ITGA2 expression levels directly correlate with their functionality. Our findings suggest that ITGA2 is a potential therapeutic target for gastric cancer.

**Electronic supplementary material:**

The online version of this article (10.1186/s12575-018-0073-x) contains supplementary material, which is available to authorized users.

## Background

Gastric cancer is the fourth most common leading cause of cancer-related death in the world, with approximately 952,000 new cases and 723,000 cancer-related deaths per year, according to the GLOBOCAN 2012 report [1]. Although surgery has been the main treatment modality for non-metastatic gastric cancer, the majority of patients diagnosed at advanced stages often unfit for surgery. The prognoses for the patients are therefore less favorable, with a 5-year survival rate lower than 30% [2–4].

Currently, monoclonal antibody has become a new venue of cancer treatments [5, 6]. Some of the antibodies such as trastuzumab (an anti-human epidermal growth factor receptor 2 (HER2) antibody) and bevacizumab (an anti-vascular endothelial growth factor (VEGF) antibody) have been used for advanced gastric cancer [7–9]. However, these therapeutic antibodies do not treat all gastric cancer patients since less than 20% of gastric cancers showed HER2 gene amplification and approximately 25% of gastric cancers showed HER2 protein overexpression [10]. Furthermore, multiple clinical trials on the efficacies of trastuzumab and bevacizumab indicate that these antibodies may not improve the patient survival over chemotherapy alone [11–13]. Therefore, identifying new markers and develop new effective and safe therapies for advanced gastric cancer is urgently needed.

ITGA2 encodes the alpha 2 integrin that may form a heterodimer with beta 1 subunit of the α2β1 integrin as a transmembrane receptor for cell adhesions to extracellular matrix (ECM) [14, 15]; alpha 2 beta1 integrin as the major collagen receptor also can bind laminin, fibronectin and E-cadherin proteins [16–19]. Moreover, several evidences have shown that α2β1 integrin is primarily expressed in newborn vessels, activated endothelial cells and immune cells in vivo [20–22]. It is also over-expressed on a variety of cancer cells, but is absent or low in most normal organs and resting endothelial cells [20, 23]. Functional studies indicate that α2β1 promotes cancer cells migration and invasion as well as angiogenesis [24–26]. In addition, numerous studies have investigated that expression profiles ofα2β1 integrin on cancer cells positively correlate the aggressive behaviors during cancer progression [27–30]. Therefore, α2 integrin may represent a promising marker for developing targeted therapeutic agents of cancers.

In this study, we found that blockade of ITGA2 by a specific antibody inhibited gastric cancer cells by two mechanisms: reduced cell viability (increased apoptosis) and reduced cell migration. The extents of inhibition positively correlated to the expression of ITGA2 as the anti-cancer effects were more pronounced in ITGA2-overexpressing cells. We further identified the mitochondrial apoptotic pathway and the actin assembly pathway as the major molecular mechanisms induced or inhibited, respectively, by the anti-ITGA2 antibody. These data suggest that ITGA2 may be a potential therapeutic target for gastric cancer.

## Methods

### Dataset for ITGA2 mRNA Expression Levels in Gastric Cancer

The mRNA data of gastric normal and tumor tissues were downloaded from The Cancer Genome Atlas (TCGA) Research Network portal (cancergenome.nih.gov). The dataset contained 32 normal gastric tissues and 375 gastric cancer tissues, which involved 27 pairs of gastric cancer tissues and their matched non-cancerous tissue samples. The total RNA-seq sample data were integrated into a matrix with log2 transformed for the downstream analysis, and listed in Additional file 1: Table S2.

Genetic characterization of 38 human gastric cancer cell lines were accessed from the Cancer Cell Line Encyclopedia (CCLE) database (portals.broadinstitute.org/ccle).

### Cell Lines and Cell Culture

The human gastric adenocarcinoma cell line AGS (ATCCno.CRL-1739) and SNU-1 cells (ATCC no. CRL-5971) were purchased from the Bioresource Collection and Research Center (Hsinchu, Taiwan). Cells were maintained in RPMI-1640 medium (Invitrogen, Carlsbad, CA, USA) supplemented with 10% fetal bovine serum (Thermo Fisher Scientific, Waltham, MA, USA), 2.2 g/L of sodium bicarbonate (Thermo Fisher Scientific) and 1% penicillin/streptomycin (Invitrogen) at 37 °C in a humidified atmosphere with 5% CO_2_.

### RNA Isolation and Reverse Transcription Polymerase Chain Reaction (RT-PCR)

The total RNA was extracted from the cells by TRIZOL reagent (Invitrogen), according to the manufacturer’s instructions. Reverse transcriptions of 5 μg total cellular RNA were performed using the SuperScript™ III One-Step RT-PCR System with Platinum™ Taq DNA Polymerase Kit (Invitrogen). Each PCR was run for 35 cycles by repeating denaturation at 95 °C for 30 s, annealing at 55 °C for 30 s, and extension at 72 °C for 1 min. The last cycle was followed by an additional incubation at 72 °C for 10 min. The sequences of the specific primers selected for the RT-PCR reactions are listed in Additional file 2: Table S1. The PCR products were size fractioned by 2% agarose gel electrophoresis and then imaged by a CCD camera. Gene expressions were presented as the relative intensities of the target RT-PCR products, which were normalized by the RT-PCR product of the glyceraldehyde 3-phosphate dehydrogenase (GAPDH) gene.

### Immunoblotting Analysis

The cells were lysed in lysis buffer (5% glycerol, 1 mM sodium EDTA, 1 mM dithiothreitol, 40 μg/ml leupeptin, 40 μg/ml aprotinin, 20 μg/ml pepstatin, 1 mM PMSF, 0.5% Triton X-100 in PBS) and then incubated on ice for 30 min. After centrifugation at 10,000 xg for 30 min at 4 °C, supernatants were collected and the protein concentrations were determined by Bradford protein assay (Bio-Rad, Hercules, CA, USA). Fifty μg of the protein lysates were resolved by 10% SDS-PAGE. The resolved proteins were transferred onto 0.2 μm PVDF membranes (PALL). After blocking the membranes with 5% skim milk in TBST buffer (10 mM Tris–HCl, pH 7.5, 150 mM NaCl, 0.05% Tween 20), a mouse anti-human ITGA2 monoclonal antibody (1:1000 dilution; Santa Cruz Biotechnology, Paso Robles, CA, USA) was added to the membranes in TBST supplemented with 5% skim milk at 4 °C overnight or at room temperature for 1 h. After extensive washing in TBST, the membranes were then probed with a goat anti-mouse peroxidase-conjugated antibody (1:10,000 dilution; Sigma–Aldrich) at room temperature for 1 h and then washed in TBST. Finally, the bound proteins were revealed by the WesternBright ECL Western blotting detection kit (Advansta, Menlo Park, CA, USA) and imaged by a CCD camera.

### Flow Cytometry for Analysis of ITGA2 Expression Levels on the Cell Surface

AGS and SNU-1 cells (2 × 10^5^) were stained by the mouse anti-human ITGA2 monoclonal antibody (1:100 dilution; Santa Cruz Biotechnology) at 4 °C for 1 h, and washed with cold 1X PBS. The cells were then incubated with a FITC-labeled goat anti-mouse IgG (H + L) antibody (1:500 dilution; Jackson Immuno Research) at 4 °C for 30 min. The antibody-treated cells were washed and resuspended in 500 μl PBS containing 1 μg/ml propidium iodide (Thermo Fisher Scientific) to distinguish live and dead cells. Cells were then analyzed by a FACS SCAN flow cytometer (BD Biosciences, San Jose, CA, USA).

### Cell Proliferation Assay

AGS and SNU-1 cells were seeded at a density of 1 × 10^4^ per well in 96-well plates overnight. The cells were treated with graded concentrations of mouse anti-human ITGA2 monoclonal antibody or isotype control antibody for another 48 h. Cell viability was measured by the 3-(4,5-dimethyl-2-thiazolyl)-2,5-diphenyl-2H-tetrazolium bromide (MTT) assay (Sigma–Aldrich, St. Louis, MO, USA).

### Analysis of Cell Apoptosis

AGS cells were seeded at a density of 1 × 10^5^ per well in 24-well plates and treated with 0.3 μg of mouse anti-human ITGA2 monoclonal antibody or isotype control for 48 h. The cells were detached by EDTA and washed cells with cold PBS. The cells were then stained by the PE Active Caspase-3 Apoptosis Kit (BD Biosciences), according to the manufacturer’s instructions. The apoptosis responses (activated caspase-3) of the treated cells were then analyzed by a FACS SCAN flow cytometer.

### Cell Migration Assay

Migration assay was performed in triplicate using a 24-well Transwell chamber system (Corning Inc., NY, USA) equipped with a filter membrane with 8 μm pores. AGS cells were seeded in the upper chamber at 1 × 10^5^ cells/well and treated with 0.1 μg of mouse anti-human ITGA2 monoclonal antibody or isotype control antibody in 0.3 ml of serum-free DMEM media. Media supplemented with 10% fetal bovine serum was placed in the bottom well in the volume of 0.5 ml. After 18 h incubation, the filter membranes were fixed with methanol and stained with 50 μg/ml propidium iodide (Thermo Fisher Scientific) for 30 min. The amount of cells that had migrated to the lower surface of the filter membrane was counted in ten random fields under an inverted fluorescence microscope (Olympus IX71, Stanford, CA, USA) at 200 × .

### Cell Morphology Analysis

AGS cells were seeded at 1 × 10^5^ on coverslips overnight (Marienfeld-Superior, Lauda-Königshofen, Germany), than the attachment of cells were treated with 0.3 μg of mouse anti-human ITGA2 monoclonal antibody or isotype control antibody for 12 h. The cells were fixed with 4% paraformaldehyde (Sigma–Aldrich) for 30 min, and permeabilized cells with 0.25% Triton X-100 (AMRESCO, Solon, OH, USA) for 15 min. After blocking with 5% BSA for 1 h, the cells were stained by FITC-conjugated phalloidin (Thermo Fisher Scientific) for 1 h. The cells were washed three times with PBS and incubated with 10 mg/ml of Hoechst 33,342 (Thermo Fisher Scientific) dye for 20 min. After another set of washes, cells were mounted onto slides with 20 μl of Dako Cytomation fluorescent mounting medium (DAKO, Glostrup, Denmark). Observation was performed in a 63× objective lens on a multiphoton and confocal microscope (Leica, Mannheim, Germany).

### Statistical Analysis

Data were analyzed using the SAS statistical software package (SAS Institute Inc., Cary, USA).The data were expressed as the means ± SD. Student’s t-test was used when comparing two independent samples and ANOVA was used when comparing multiple samples. Differences with *p* < 0.05 were considered statistically significant.

## Results

### Expressions of ITGA2 in Gastric Cancer Cell Lines

**Fig. 1:**
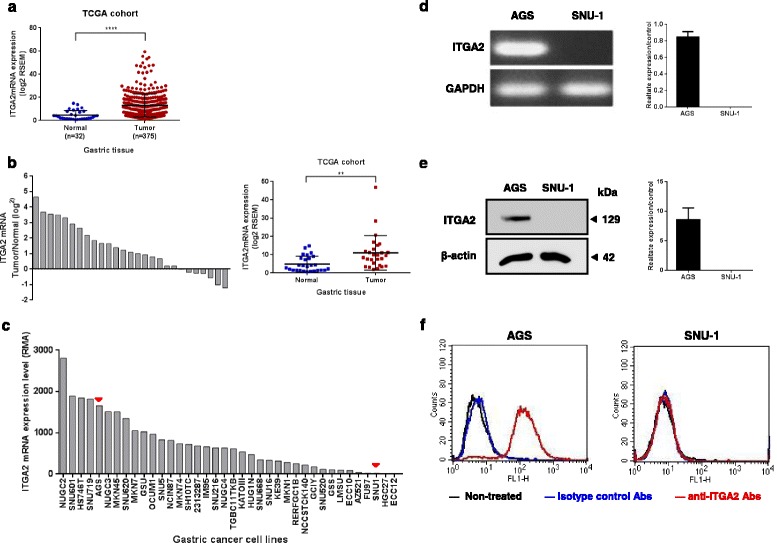
Analysis of ITGA2 expression levels in gastric cancers. **a** Data of ITGA2 mRNA levels were extracted from 32 normal gastric tissue and 375 gastric cancer tissue in the TCGA datasets. The result was expressed as mean ± standard deviation (SD), and statistical comparisons were made by Wilcoxon signed rank test. **** *p* < 0.0001. **b** The mRNA levels of ITGA2 in 27 available paired gastric normal and tumor tissues were compared, and a positive log^2^ (tumor/normal) value indicates increased expression, while a negative log^2^ value indicates decreased expression, of ITGA2 in the gastric tumor tissues. ITGA2 mRNA was significantly overexpressed in gastric tumor tissues as compared with normal tissues (*p* < 0.01, paired *t-test*). Result was expressed as mean ± standard deviation (SD). **c** mRNA expression profiles for ITGA2 across gastric cancer cell lines in the Cancer Cell Line Encyclopedia (CCLE) database. Red arrowheads mark the expression levels of AGS and SUN-1 cells. **d** The expression of ITGA2 mRNA was measured by quantitative real-time PCR in AGS and SNU-1 cells. Glyceraldehyde-3-phosphate dehydrogenase (GAPDH) was used as an internal control. **e** The ITGA2 protein expression was semi-quantified by western blot analyses. **d** and **e** The densitometric measurements of ITGA2 in AGS and SNU-1 cells were normalized to the internal control (β-actin). **f** The surface expressions of ITGA2 were determined on PI-negative cells, using flow cytometry. Data are representative of three independent experiments

To determine whether ITGA2 was over-expressed in gastric cancers, mRNA expression of ITGA2 in 32 normal gastric tissues and 375 gastric cancer tissues from the Caner Genome Atlas project (TCGA) were investigated. As shown in Fig. 1a, the mRNA expressions of ITGA2 were significantly higher in gastric cancers than in the normal gastric tissues (cancer expression (mean ± SD): 13.1 ± 10 vs. normal expression: 4.6 ± 3.98, *p* < 0.0001). To compare the expression of ITGA2 in gastric cancer and normal tissue in a same patient, 27 pairs of gastric cancer tissues and their matched non-cancerous tissue samples were compared. ITGA2 was found to be over-expressed in 74% (20/27) of the patients (Fig. 1b). Statistical analysis using the paired *t-test* indicated a significant difference in the mRNA levels of ITGA2 between the gastric cancer and non-cancerous tissues (cancer expression: 11.1 ± 9.39 vs. normal expression: 4.9 ± 4.24, *p* < 0.01). These data indicate that ITGA2 may play important role(s) in gastric cancer development.

Additionally, the ITGA2 mRNA expression levels of 38 human gastric cancer cell lines were analyzed from the Cancer Cell Line Encyclopedia (CCLE) database. A spectrum of ITGA2 expression was noted across the gastric cancer cell lines studied. Seven human gastric cancer cell lines expressed high (defined as RMA > mean + 1 SD) and 5 human gastric cancer cell lines expressed low (defined as robust multi-array average (RMA) < mean-1 SD) ITGA2 mRNA levels (Fig. 1c), among which the AGS cells expressed high level of ITGA2 (RMA 1656.7) and the SUN-1 cells express low level of ITGA2 (RMA 18.0). We chose AGS and SUN-1 for further verifications by RT-PCR (normalized AGS expression: 0.9 ± 0.06 vs. normalized SUN-1 expression: non-detected, *p* < 0.0001, Fig. 1d) and western blot (normalized AGS expression: 8.6 ± 1.94 vs. SUN-1 expression: non-detected, *p* < 0.01, Fig. 1e). From these results, it is clear that AGS expressed high levels of mRNA and proteins of ITGA2, whereas ITGA2 expression in SUN-1 was low or undetectable. Furthermore, ITGA2 was expressed on the plasma membrane in AGS cells, but no expression on SNU-1 cells was noted (AGS mean fluorescence intensity (MFI): 178.7 vs. SUN-1 MFI: 4.2, p < 0.0001, Fig. 1f). Thus, we confirmed that the AGS and SNU-1 cells display contrasting ITGA2 expressions and we used these two cell lines to further investigate the potential role of ITGA2 in gastric cancer.

### Anti-ITGA2 Antibody Induced Apoptosis in ITGA2-Overexpressed Gastric Cancer Cells

**Fig. 2:**
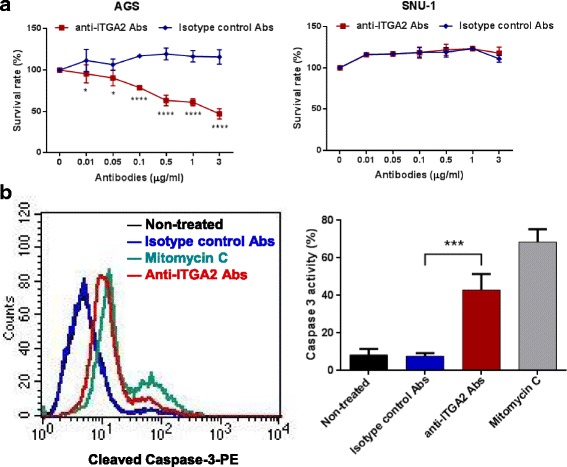
The anti-ITGA2 antibody induced apoptosis in AGS cells but not SUN-1 cells. **a** Human gastric cancer cell lines AGS and SUN-1 were treated with different concentrations of an anti-ITGA2 antibody for 48 h, and cell survivals were measured by an MTT assay. An isotype control antibody was used as a negative control. **b** AGS cells were treated with mitomycin C as a positive control, or incubated with 0.3 μg of anti-ITGA2 antibodies or isotype control (negative control) for 48 h. Cells were stained with PE-conjugated anti-active caspase-3 antibodies, and analyzed by flow cytometry. The percentages of caspase-3 positive-staining cells summarized in the right panel, showing mean ± standard deviation (S.D). Statistical comparisons were made by one-way ANOVA or two-way ANOVA with Bonferroni comparisons. * *p* < 0.05 and **** *p* < 0.0001. Data are representative of three independent experiments

To investigate whether blocking ITGA2 could affect survival of gastric cancer cells, AGS and SNU-1 cells were treated with graded concentrations of an anti-ITGA2 antibody or an isotype control antibody and examined for cell numbers by an MTT assay. The anti-ITGA2 antibody dose-dependently inhibited the cell proliferation in AGS cells (Fig. 2a). The anti-ITGA2 antibody could inhibit AGS cells, even at low doses (0.01 μg/ml), albeit effect was minimal. Higher doses of the anti-ITGA2 antibody more efficiently inhibited the survival (~ 50% inhibition at 3 μg/ml) of the AGS cells. On the contrary, the anti-ITGA2 antibody did not cause any inhibition in SNU-1 cells. The anti-ITGA2 antibodies caused membrane shrinkage and apoptotic body-like structures in anti-ITG2 antibody-treated AGS cells (Additional file 3: Figure S1). Thus, we investigated whether anti-ITGA-2 antibody induce apoptosis in AGS cells. The levels of active caspase-3 were significantly increased in AGS cells treated with anti-ITGA2 antibodies compared to isotype control (anti-ITGA2 antibody MFI: 19.3 vs. isotype control MFI: 7.4, *p* < 0.001, Fig. 2b). These results suggested that blocking ITGA2 by a specific antibody induces apoptosis in ITGA2-overexpressing cells.

### ITGA2 Blockade Up-Regulated Bim-Mediated Mitochondrial Apoptotic Pathway

**Fig. 3:**
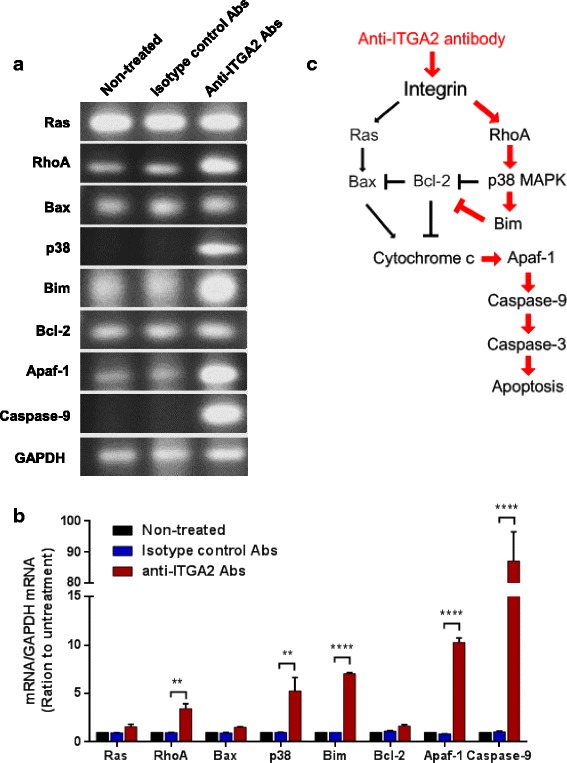
The anti-ITGA2 antibody induced a RhoA-p38 MAPK-mediated apoptotic pathway in AGS cells. **a** mRNA expression of Ras, RhoA, Bax, p38, Bim, Bcl-2, Apaf-1 and Caspase-9 in AGS cells treated with 0.3 μg of anti-ITGA2 antibodies or isotype control antibodies (negative control) for 48 h were analyzed by RT-PCR. Each analysis was derived from the same experiment and gels were processed in parallel. Cropped gels are displayed to compare gene expressions in different treatment groups. **b** Densitometric measurements on the intensities of each RT-PCR product was normalized to the mRNA level of GAPDH, and displayed as mean ± standard deviation (S.D). Statistical comparisons were made by two-way ANOVA with Bonferroni comparisons. ** *p* < 0.01, **** p < 0.0001. Data are representative of three independent experiments. **c** Summary of the anti-ITGA2 antibody-mediated apoptosis signaling pathway in AGS gastric cancer cells

To characterize the molecular mechanism by which blockade of ITGA2 induced apoptosis in gastric cancer cells, we analyzed the mRNA expression of genes that are involved in the apoptotic pathways. RhoA and p38 MAPK mRNAs were strongly up-regulated in AGS cells after treatment by the anti-ITGA2 antibody, consistent with previous findings that activated RhoA-p38 pathway may induce apoptosis [31, 32]. Expression of the pro-apoptosis factor Bim was highly up-regulated in AGS cells after prolonged exposure to anti-ITGA2 antibody (anti-ITGA2 antibody: 7.1 ± 0.11 vs. isotype control: 1.0 ± 0.01, *p* < 0.0001), whereas expression of the anti-apoptotic factor Bcl-2 did not change (Fig. 3a and b). Interestingly, expression of the other pro-apoptotic factor Bax and anti-apoptotic factor Bcl-2 were unaltered by the anti-ITGA2 antibody treatment. Bim up-regulation can trigger cytochrome c release from mitochondria and induce the formation of the apoptosome (Apaf1 and cytochrome c) that led to activation of Caspase-9 and apoptosis. Thus, we analyzed the expression of Apaf-1 and Caspase-9 expression. ITGA2 blockade strongly induced Apaf-1 (anti-ITGA2 antibody: 10.3 ± 0.45 vs. isotype control: 0.8 ± 0.01, p < 0.0001) and Caspase-9 (anti-ITGA2 antibody: 87.2 ± 9.44 vs. isotype control: 1.1 ± 0.07, p < 0.0001) expression (Fig. 3a and b). These results indicate that ITGA2 blockade induces apoptosis through Bim, cytochrome c/Apaf-1 and Caspase-9, but not through Bcl-2 or Bax.

### ITGA2 Blockade Reduced Migration of ITGA2-Overexpressing Gastric Cancer Cells

**Fig. 4:**
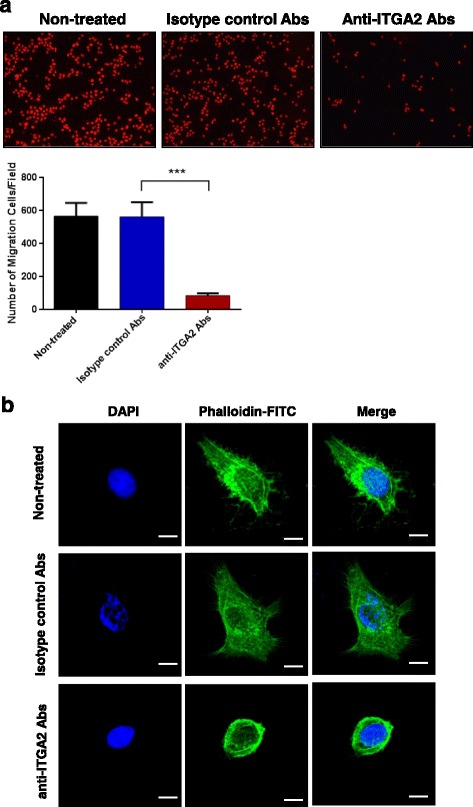
Blockade of ITGA2 reduced migration of AGS cells. **a** AGS cells were treated with 0.1 μg anti-ITGA2 antibodies or isotype control antibodies (negative control) for 18 h. Cells in the lower face of transwell membranes were stained by PI and imaged (upper panel) and data summarized as mean ± standard deviation (S.D) (lower panel). Statistical comparisons were made by two-way ANOVA with Bonferroni comparisons. *** *p* < 0.001. **b** AGS cells were treated with 0.3 μg of the anti-ITGA2 antibody for 12 h. F-actin (green) was stained with FITC-conjugated phalloidin, and nuclei stained by DAPI. Scale bar = 10 μm. Data are representative of three independent experiments

Integrins are well known for regulating cell mobility through actin rearrangement. Thus we performed transwell cell migration assay to determine whether ITGA2 blockade could inhibit migration of gastric cancer cells. Since high dosage and prolonged incubation of the anti-ITGA2 antibody induced apoptosis in ITGA2-overexpressing AGS cells, we used a non-cytotoxic dosage of the anti-ITGA2 antibody (0.1 μg/ml, Additional file 4: Figure S2) and shorter exposure time (18 h) to study the effect of ITGA2 blockade on cell migration. As expected, low dose of anti-ITGA2 antibody treatments reduced the cells that migrated through the membrane by ~ 6.6 fold, as compared to the isotype control treatments (Fig. 4a). Decreased cell migration of AGS cells was accompanied by morphological alterations in the anti-ITGA2 antibody treated cells (Fig. 4b). *Lamellipodia* and filopodia that correlated with cell migration were markedly reduced and cells rounded up upon treatment with anti-ITGA2 antibodies. The confocal microscopy and image analysis revealed a ring-like actin structure, indicating disruption of F-actin formation, induced by low dose anti-ITGA2 antibodies. These findings suggested that ITGA2 blockade inhibit cell migration through destabilizing F-actin.

### ITGA2 Blockade Reduced Actin Polymerization through the Rac1/CDC42 Signaling Pathway

**Fig. 5:**
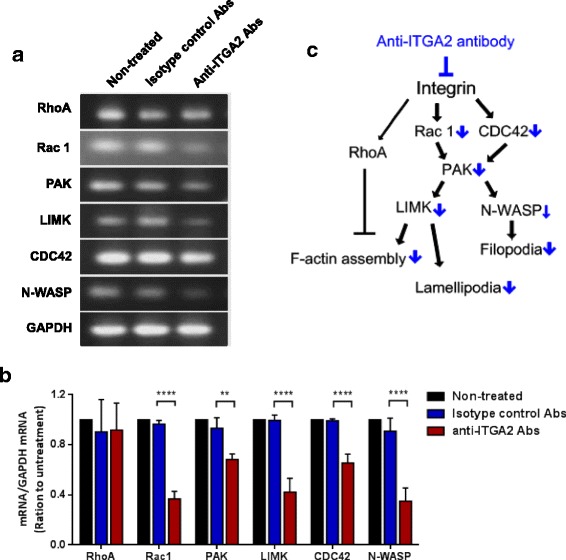
Anti-ITGA2 antibody reduced cell migration of AGS cells through down-regulation of Rac1 and CDC42 signaling pathway. **a** Gene expression of LIMK, Rac1, PAK, CDC42 and N-WASP in AGS after treatment with 0.1 μg anti-ITGA2 antibodies or isotype control antibodies (negative control) for 18 h. Each analysis was derived from the same experiment and gels were processed in parallel. Cropped gels are displayed for comparison between gene expressions in different groups. **b** Densitometric measurements on the intensities of each RT-PCR product was normalized to the mRNA level of GAPDH, and displayed as mean ± standard deviation (S.D). Statistical comparisons were made by two-way ANOVA with Bonferroni comparisons. ** *p* < 0.01 and **** *p* < 0.0001. **c** Summary of the anti-ITGA2 antibody-mediated migration signaling pathway in AGS gastric cancer cells

To investigate how ITGA2 blockade may affect F-actin turnover, we examined the expression of RhoA, Rac1, CDC42, PAK, LIMK and N-WASP in anti-ITGA2 antibody treated AGS cells. mRNA expression of RhoA (anti-ITGA2 antibody: 0.9 ± 0.22 vs. isotype control: 0.9 ± 0.26), Rac1 (anti-ITGA2 antibody: 0.4 ± 0.06 vs. isotype control: 1.0 ± 0.03, *p* < 0.0001), CDC42 (anti-ITGA2 antibody: 0.6 ± 0.07 vs. isotype control: 1.0 ± 0.01, p < 0.0001), PAK (anti-ITGA2 antibody: 0.7 ± 0.45 vs. isotype control: 0.9 ± 0.08, *p* < 0.01), LIMK (anti-ITGA2 antibody: 0.4 ± 0.11 vs. isotype control: 1.0 ± 0.04, *p* < 0.0001) and N-WASP (anti-ITGA2 antibody: 0.4 ± 0.45 vs. isotype control: 0.9 ± 0.11, *p* < 0.0001) were markedly reduced in anti-ITGA2 antibodies treated cells (Fig. 5a and b). These results suggested that ITGA2 blockade down-regulated Rac 1 and CDC42 to inhibit actin filament assembly and impeded filopodia formation and lamellipodial protrusion (Fig. 5c). Accordingly, ITGA2 signaling inhibition may prevent metastasis of gastric cancer through down-regulating Rac 1/CDC42 signaling pathway.

## Discussion

Gastric cancer has few or no symptoms in the early stages thus ~ 65% patients present as advanced cancers (T3, T4) [33, 34]. Treatment outcomes for gastric cancer (by cisplatin/fluoropyrimidine or by trastuzumab in combination with chemotherapy for HER2-positive advanced gastric cancer) are unsatisfying; the 5-year survival rate of patients with advanced gastric cancer is below 20% and has not been improved in recent 20 years [35–37]. Although several chemical drugs (such as taxane and irinotecan [38]) or monoclonal antibodies (such as pembrolizumab and ramucirumab [39, 40]) are currently tested in phase II and III clinical trials, increasing reports indicates that these drugs do not significantly improve the therapeutic outcome over conventional chemotherapy [41–44] and the prognosis remains poor. To address this important issue, our lab is dedicated to finding new molecule candidates for the developments of targeted therapies. Our data indicated that ITGA2 may represent a promising candidate because a majority (74%) of gastric cancers over-expressed ITGA2. Moreover, blockade of ITGA2 by a specific antibody dually suppresses gastric cancer by inhibiting cell migration and inducing apoptosis. Thus the development of targeted therapies against ITGA2 may improve survival of advanced gastric cancer patients.

Recent studies showed that the ITGA2 gene over-expression is associated with increased risks of prostate cancer, breast cancer and gastric cancer [45–47]. Up-regulation of ITGA2 seems to be one of the important factors accelerating tumor progression and metastasis in various types of cancers, including gastric cancer [22, 48, 49]. In line with this notion, blocking ITGA2 was shown to inhibit cell migration and metastasis of gastric cancer [50, 51]. Surprisingly, we found that prolonged blockade of ITGA2 by a specific antibody at a relatively low dose (3 μg/ml) also suppresses gastric cancer cell survival, which is previously unknown. Blockade of ITGA2 by the antibody may induce apoptosis through RhoA-p38 MAPK signaling pathway in gastric cancer cells, consistent with previous reports that the RhoA and p38 MAPK are rapidly activated, upon cellular cholesterol depletion, to induce apoptosis [31]. In addition, RhoA and p38 MAPK activation is responsible for simvastatin-induced apoptosis in osteosarcoma cells [32]. However, other reports suggest RhoA activation may lead to cell death via activation of JNK-dependent signaling pathways and activation of pro-apoptotic protein Bim [52–54]. We did not address whether JNK pathways have involved the gastric cancer cell death treated with the anti-ITGA2 antibody at the moment. Nevertheless, further studies are needed to better delineate the mechanism for developing a targeted therapy for gastric cancer.

In this study, we found that caspase-3 expression was increased in AGS gastric cancer cells after treatment with the anti-ITGA2 antibody. Furthermore, we identified that RhoA, p38 MAPK and Bim were remarkably up-regulated by anti-ITGA2 antibody. This pathway against the activity of Bcl-2, the anti-apoptotic element, will facilitate apoptosis. Moreover, the increased Apaf-1, Caspase-9 and 3 will further activate the mitochondrial apoptotic death pathway (Fig. 3c). The molecular mechanisms underlying its apoptosis effects are incompletely understood, however, the intracellular (mitochondrial) apoptotic pathway is most likely involved. Bim, Apaf-1 and Caspase-9 were markedly up-regulated by anti-ITGA2 antibody, whereas Bcl-2 and Bax were unaltered. It is well known that Bim activates apoptosis by binding to Bcl-2 and disrupt Bcl-2-Bax complexes to release Bax. Free Bax then forms homodimer, or forms heterodimer with Bak, to subsequently promote cytochrome c release from the mitochondria [55–59]. Cytosolic cytochrome c form complex with Apaf-1 to activate caspase-9 which in turn activates caspase-3. Thus, although the expression of Bax and Bcl-2 were unaltered by the ITGA2 blockade, increased Bim expression is expected to promote Bax homo- or heterodimer formations to promote induction of apoptosis. On the other hand, although p38 MAPK was shown to mediate apoptosis by down-regulating anti-apoptotic Bcl-2 family proteins (e.g. Bcl-2, Bcl-XL) [60–62] and up-regulating the pro-apoptotic Bcl-2 family proteins (e.g. Bax, Bid and Bim) [54, 63, 64], RhoA-p38 MAPK pathway do not seem to regulate Bcl-2 and Bax expression in the case of ITGA2 blockade.

It is well established that F-actin assembly is necessary for protrusion dynamics and shaping cell morphology as well as cell migration [65–68]. In addition, epithelial-mesenchymal transition (EMT), a process where epithelial cells transformed into mesenchymal phenotypes, depends on the reorganization of cytoskeletal structures, which result in cell shape changes, cell elongation and membrane protrusions promote gastric cancer aggressiveness [69–71]. Integrins can either promote or suppress actin cytoskeleton with mediated cellular signaling pathway involved in cell movement and function [72–74]. We show that block ITGA2 has a dramatic impact on cell morphology in AGS cells. Furthermore, we found that a non-cytotoxic dose of the anti-ITGA2 antibody in shorter exposure reduced significantly migration of AGS cells, but had no effect on total cell number. In addition, our results indicated that blocking ITGA2 significantly decreased Rac 1 and CDC42 expressions, which further reduced the expressions of PAK. Reduced PAK signaling might also decrease the expressions of both N-WASP and LIMK, which impaired the filopodia formation, F-actin filament assembly and lamellipodial protrusion (Fig. 5c). These results are consistent with previous studies that CDC42 and Rac 1 are involved in intracellular signaling pathways downstream of integrins and affected actin filament organization [75–78]. For instance, modulation of CDC42 interaction with N-WASP has been shown to be implicated in filopodia formation [79, 80] and Rac1 recognized as a key regulator in the formation of lamellipodia [81, 82]. Thus, blocking ITGA2 is expected to eliminate both filopodia and lamellipodia formation by down-regulate Rac1 and Cdc42. It is also interesting to speculate whether blockade of ITGA2 can inhibit EMT since the small Rho GTPases, Rho, Rac and CDC42, have been shown to promote the EMT transition via down-regulated expression of E-cadherin and rearrange actin cytoskeleton that drive cell polarity and invasiveness [76, 83, 84]. Collectively, these data also support that blocking ITGA2 may be a promising method to inhibit migration of gastric cancers.

The integrin α2 is expressed in epithelial cells, platelets, megakaryocytes and fibroblasts [20–22]. Direct application of anti-ITGA2 antibodies to treat gastric cancer may induce considerable cytotoxicity to these cells. Several strategies may be used to reduce the risks of this complication and improve the safety of the antibodies, including designing pro-antibodies by masking the binding sites of antibodies which may cleaved by proteases that highly expressed at tumor sites [85–87]. This strategy may direct the therapeutic activity of the anti-ITGA2 antibodies to tumor lesions, but not the normal tissues. This development may alleviate the cytotoxicity associated with the antibody to achieve a better therapeutic index for gastric cancers.

## Conclusions

In this study, our results demonstrated that ITGA2 is over-expressed in a majority of gastric cancer. Blockade of ITGA2 dually inhibited gastric cancer cells by inducing apoptosis and inhibiting cell migration, depending on the dose and duration of exposure. Notably, we showed that the RhoA-p38 MAPK signaling pathway promoted up-regulation of Bim to elicit the cytochrome c release, and following Apaf-1, Caspase-9 and 3 activated to induce the mitochondrial apoptotic death pathway, whereas the expression of Ras and Bax/Bcl-2 was not affected. We also discussed the down-regulation of N-WASP, PAK and LIMK, downstream of Cdc42 and Rac1, which altered actin organization and was involved in targeting ITGA2 inhibited cell migration of gastric cancer cells. Thus, targeting ITGA2 may be a promising therapy to improve the survival of gastric cancer patients.

## Additional files

Additional file 1: **Table S2.** RNA-seq sample annotation for each of the 32 normal gastric tissues and 375 gastric cancer tissues from TCGA. (DOCX 16 kb)

Additional file 2: **Table S1.** Primer list for reverse transcription-polymerase chain reactions (RT-PCR). (DOCX 15 kb)

Additional file 3: **Figure S1.** Effect of anti-ITGA2 antibody on cell morphology. The AGS cells were treated with a 3 μg of the anti-ITGA2 antibodies or isotype control antibodies (negative control) for 48 h, and cell morphology was observed at 200X magnification. Data are representative of three independent experiments. (PPTX 1463 kb)

Additional file 4: **Figure S2.** Low dose of anti-ITGA2 antibodies did not induce cell death in AGS cells. Photography and quantitative analyses on cell number of the AGS cells treated with 0.1 μg anti-ITGA2 antibodies or isotype control antibodies (negative control) for 18 h. Data are expressed as mean ± standard deviation (S.D). Statistical comparisons were made by one-way ANOVA with Bonferroni comparisons. Data are representative of three independent experiments. (PPTX 784 kb)

## Abbreviations

ECM: Extracellular matrix
HER2: Human epidermal growth factor receptor 2
ITGA2: Integrin alpha 2
RT-PCR: Reverse transcription polymerase chain reaction
VEGF: Vascular endothelial growth factor
