# Supplementary material for: Blockade of ITGA2 Induces Apoptosis and Inhibits Cell Migration in Gastric Cancer
Source: Biol Proced Online. 2018 May 1;20:10. doi: 10.1186/s12575-018-0073-x (PMC5928594; doi:10.1186/s12575-018-0073-x)
Supplement: Supplementary file 1 — Table S2. RNA-seq sample annotation for each of the 32 normal gastric tissues and 375 gastric cancer tissues from TCGA. (DOCX 16 kb) [file 12575_2018_73_MOESM1_ESM.docx]

**Table S2.** RNA-seq sample annotation for each of the 32 normal gastric tissues and 375 gastric cancer tissues from TCGA.

| **Normal gastric tissues** | TCGA-HU-A4GY-11A-11R-A36D-31,TCGA-BR-6453-11A-01R-1802-13,TCGA-BR-7715-11A-01R-2055-13,TCGA-CG-5720-11A-01R-1602-13,TCGA-BR-6802-11A-01R-1884-13,TCGA-CG-5734-11A-01R-1602-13,TCGA-HU-A4GN-11A-12R-A251-31,TCGA-IN-8663-11A-01R-2402-13,TCGA-IN-AB1X-11A-21R-A39E-31,TCGA-CG-5733-11A-01R-1602-13,TCGA-FP-7829-11A-01R-2055-13,TCGA-BR-8060-11A-01R-2343-13,TCGA-HU-A4HB-11A-11R-A251-31,TCGA-CG-5728-11A-01R-1602-13,TCGA-HU-A4GC-11A-11R-A251-31,TCGA-BR-6454-11A-01R-1802-13,TCGA-CG-5721-11A-01R-1602-13,TCGA-BR-7716-11A-01R-2055-13,TCGA-HU-A4GP-11A-21R-A251-31,TCGA-BR-7851-11A-01R-2203-13,TCGA-CG-5730-11A-01R-1602-13,TCGA-IP-7968-11A-01R-2203-13,TCGA-BR-7704-11A-01R-2055-13,TCGA-CG-5722-11A-02R-1602-13,TCGA-IN-7806-11A-01R-2055-13,TCGA-BR-7703-11A-01R-2055-13,TCGA-IN-AB1V-11A-11R-A414-31,TCGA-HU-8238-11A-01R-2343-13,TCGA-HU-A4GH-11A-11R-A36D-31,TCGA-BR-7717-11A-01R-2055-13,TCGA-BR-6457-11A-01R-1802-13,TCGA-FP-7735-11A-01R-2055-13 |
| --- | --- |
| **Gastric cancer tissues** | TCGA-HU-A4G3-01A-11R-A24K-31,TCGA-D7-8570-01A-11R-2343-13,TCGA-VQ-A8P2-01A-11R-A36D-31,TCGA-BR-8687-01A-11R-2402-13,TCGA-CD-5801-01A-11R-1602-13,TCGA-VQ-AA64-01A-11R-A414-31,TCGA-D7-6528-01A-11R-1802-13,TCGA-CG-5724-01A-11R-1602-13,TCGA-HF-7134-01A-11R-2055-13,TCGA-VQ-A91Z-01A-11R-A414-31,TCGA-CD-8528-01A-11R-2343-13,TCGA-BR-6566-01A-11R-1802-13,TCGA-VQ-A91V-01A-11R-A414-31,TCGA-CG-5717-01A-11R-1602-13,TCGA-CG-4466-01A-01R-1157-13,TCGA-BR-7959-01A-11R-2343-13,TCGA-CG-4476-01A-01R-1157-13,TCGA-D7-8576-01A-11R-2343-13,TCGA-CG-5720-01A-11R-1602-13,TCGA-CD-8525-01A-11R-2343-13,TCGA-CD-5803-01A-11R-1602-13,TCGA-BR-8485-01A-11R-2402-13,TCGA-CD-8530-01A-11R-2343-13,TCGA-BR-6456-01A-11R-1802-13,TCGA-VQ-A8PJ-01A-11R-A414-31,TCGA-VQ-AA6F-01A-31R-A414-31,TCGA-BR-A4CS-01A-11R-A24K-31,TCGA-HJ-7597-01A-21R-2203-13,TCGA-CD-5800-01A-11R-1602-13,TCGA-HU-8604-01A-11R-2402-13,TCGA-VQ-A8DV-01A-12R-A36D-31,TCGA-VQ-A94R-01A-11R-A414-31,TCGA-BR-7196-01A-11R-2055-13,TCGA-HU-A4H8-01A-11R-A251-31,TCGA-CG-5732-01A-11R-1602-13,TCGA-BR-8367-01A-11R-2343-13,TCGA-VQ-A91K-01A-11R-A414-31,TCGA-CD-5799-01A-11R-1602-13,TCGA-R5-A7ZR-01A-11R-A354-31,TCGA-VQ-A8PQ-01A-11R-A414-31,TCGA-IN-A6RJ-01A-21R-A33Y-31,TCGA-B7-A5TK-01A-12R-A36D-31,TCGA-IN-8663-01A-11R-2402-13,TCGA-FP-8099-01A-11R-2343-13,TCGA-CD-8535-01A-11R-2343-13,TCGA-MX-A666-01A-11R-A31P-31,TCGA-BR-8592-01A-11R-2402-13,TCGA-BR-8682-01A-11R-2402-13,TCGA-D7-A6EZ-01A-11R-A31P-31,TCGA-VQ-A924-01A-11R-A414-31,TCGA-HU-A4G9-01A-11R-A24K-31,TCGA-VQ-A8PH-01A-12R-A414-31,TCGA-BR-4187-01A-01R-1131-13,TCGA-BR-8486-01A-31R-2402-13,TCGA-D7-A74A-01A-11R-A32D-31,TCGA-CG-5721-01A-11R-1602-13,TCGA-ZQ-A9CR-01A-11R-A39E-31,TCGA-VQ-A91X-01A-12R-A414-31,TCGA-IP-7968-01A-11R-2203-13,TCGA-D7-6521-01A-11R-1802-13,TCGA-BR-6852-01A-11R-1884-13,TCGA-VQ-A91A-01A-11R-A414-31,TCGA-IN-8462-01A-11R-2343-13,TCGA-HU-A4GU-01A-11R-A251-31,TCGA-EQ-8122-01A-11R-2343-13,TCGA-CD-A4MH-01A-11R-A251-31,TCGA-D7-6815-01A-11R-1884-13,TCGA-BR-8060-01A-11R-2343-13,TCGA-D7-8578-01A-21R-2343-13,TCGA-BR-4201-01A-01R-1131-13,TCGA-D7-A6F2-01A-12R-A31P-31,TCGA-BR-7901-01A-11R-2203-13,TCGA-F1-6177-01A-11R-1802-13,TCGA-HU-8249-01A-11R-A36D-31,TCGA-BR-7851-01A-11R-2203-13,TCGA-HU-A4HD-01A-11R-A251-31,TCGA-VQ-A8PO-01A-11R-A414-31,TCGA-3M-AB46-01A-11R-A414-31,TCGA-HU-A4G8-01A-11R-A251-31,TCGA-VQ-A923-01A-11R-A414-31,TCGA-VQ-A91D-01A-11R-A414-31,TCGA-HU-A4GT-01A-21R-A251-31,TCGA-BR-4370-01A-01R-1157-13,TCGA-BR-8365-01A-21R-2343-13,TCGA-BR-6801-01A-11R-1884-13,TCGA-CD-8531-01A-11R-2343-13,TCGA-R5-A7ZF-01A-11R-A354-31,TCGA-IN-AB1V-01A-21R-A414-31,TCGA-FP-8209-01A-11R-2343-13,TCGA-BR-8080-01A-11R-2343-13,TCGA-VQ-A925-01A-11R-A414-31,TCGA-CG-5716-01A-21R-1802-13,TCGA-HU-8602-01A-11R-2402-13,TCGA-BR-8296-01A-11R-2343-13,TCGA-D7-A6F0-01A-11R-A31P-31,TCGA-D7-5577-01A-01R-1602-13,TCGA-VQ-A94T-01A-11R-A414-31,TCGA-D7-6818-01A-11R-1884-13,TCGA-KB-A6F7-01A-12R-A32D-31,TCGA-R5-A7ZI-01A-11R-A354-31,TCGA-VQ-A91Q-01A-12R-A414-31,TCGA-BR-4253-01A-01R-1131-13,TCGA-BR-4294-01A-01R-1131-13,TCGA-VQ-A8P3-01A-11R-A36D-31,TCGA-RD-A7BW-01A-11R-A32D-31,TCGA-CD-5798-01A-11R-1602-13,TCGA-BR-4371-01A-01R-1157-13,TCGA-BR-A4J7-01A-31R-A251-31,TCGA-VQ-A8P5-01A-11R-A39E-31,TCGA-CG-4305-01A-01R-1157-13,TCGA-IN-A7NT-01A-21R-A354-31,TCGA-BR-4267-01A-01R-1131-13,TCGA-CG-5725-01A-11R-1602-13,TCGA-D7-6527-01A-11R-1802-13,TCGA-CG-4304-01A-01R-1157-13,TCGA-BR-7717-01A-11R-2055-13,TCGA-BR-7957-01A-11R-2203-13,TCGA-VQ-A8PD-01A-11R-A414-31,TCGA-VQ-A8P8-01A-11R-A39E-31,TCGA-HU-8608-01A-11R-2402-13,TCGA-F1-6874-01A-11R-1884-13,TCGA-BR-6709-01A-11R-1884-13,TCGA-BR-A4J8-01A-11R-A251-31,TCGA-BR-A4PF-01A-11R-A251-31,TCGA-HU-A4G2-01A-11R-A251-31,TCGA-F1-A448-01A-11R-A24K-31,TCGA-BR-8690-01A-11R-2402-13,TCGA-CD-8529-01A-11R-2343-13,TCGA-D7-A6EX-01A-11R-A31P-31,TCGA-CG-4477-01A-01R-1157-13,TCGA-BR-7197-01A-11R-2203-13,TCGA-HU-A4HB-01A-12R-A251-31,TCGA-CD-A4MG-01A-11R-A251-31,TCGA-BR-8686-01A-11R-2402-13,TCGA-BR-8590-01A-11R-2402-13,TCGA-BR-4257-01A-01R-1131-13,TCGA-HU-A4GQ-01A-11R-A36D-31,TCGA-CG-5723-01A-11R-1602-13,TCGA-VQ-A94P-01A-13R-A414-31,TCGA-BR-8677-01A-11R-2402-13,TCGA-CD-8524-01A-11R-2343-13,TCGA-CG-4438-01A-01R-1157-13,TCGA-HU-A4H4-01A-21R-A251-31,TCGA-BR-8589-01A-11R-2402-13,TCGA-BR-8683-01A-11R-2402-13,TCGA-HU-A4GX-01A-12R-A251-31,TCGA-BR-8372-01A-11R-2343-13,TCGA-BR-8361-01A-11R-2343-13,TCGA-VQ-A8E2-01A-11R-A36D-31,TCGA-BR-4369-01A-01R-1157-13,TCGA-VQ-A8PM-01A-21R-A414-31,TCGA-D7-8572-01A-11R-2343-13,TCGA-BR-A4J5-01A-21R-A251-31,TCGA-BR-8484-01A-11R-2402-13,TCGA-BR-4361-01A-01R-1157-13,TCGA-CG-4444-01A-01R-1157-13,TCGA-3M-AB47-01A-22R-A414-31,TCGA-D7-6526-01A-11R-1802-13,TCGA-B7-A5TJ-01A-11R-A31P-31,TCGA-FP-A8CX-01A-11R-A36D-31,TCGA-D7-5578-01A-01R-1602-13,TCGA-RD-A8N2-01A-12R-A36D-31,TCGA-HU-A4GD-01A-11R-A36D-31,TCGA-BR-6452-01A-12R-1802-13,TCGA-CG-4441-01A-01R-1802-13,TCGA-CG-4440-01A-01R-1157-13,TCGA-BR-8286-01A-12R-2343-13,TCGA-RD-A8N9-01A-12R-A39E-31,TCGA-F1-6875-01A-11R-2055-13,TCGA-IN-A6RI-01A-11R-A32D-31,TCGA-BR-8680-01A-11R-2402-13,TCGA-D7-A4YU-01A-21R-A251-31,TCGA-VQ-A8PX-01A-12R-A414-31,TCGA-BR-8678-01A-11R-2402-13,TCGA-VQ-A91S-01A-11R-A414-31,TCGA-MX-A663-01A-11R-A31P-31,TCGA-RD-A8N6-01A-11R-A36D-31,TCGA-BR-7715-01A-11R-2055-13,TCGA-BR-8591-01A-11R-2402-13,TCGA-FP-A9TM-01A-11R-A39E-31,TCGA-B7-A5TI-01A-11R-A31P-31,TCGA-VQ-AA6J-01A-11R-A414-31,TCGA-BR-8371-01A-11R-2343-13,TCGA-BR-4363-01A-01R-1157-13,TCGA-RD-A8N1-01A-12R-A36D-31,TCGA-CD-5813-01A-11R-1602-13,TCGA-HF-A5NB-01A-11R-A31P-31,TCGA-BR-6453-01A-11R-1802-13,TCGA-BR-6455-01A-11R-1802-13,TCGA-IN-A7NU-01A-22R-A354-31,TCGA-BR-8297-01A-12R-2343-13,TCGA-CG-4475-01A-01R-1157-13,TCGA-CG-4462-01A-01R-1157-13,TCGA-BR-6563-01A-13R-2055-13,TCGA-MX-A5UJ-01A-11R-A31P-31,TCGA-BR-7723-01A-11R-2055-13,TCGA-BR-4368-01A-01R-1157-13,TCGA-D7-A4YX-01A-11R-A251-31,TCGA-BR-4279-01A-01R-1131-13,TCGA-HU-A4H5-01A-21R-A251-31,TCGA-BR-7716-01A-21R-2055-13,TCGA-BR-A4J9-01A-12R-A251-31,TCGA-VQ-A8PK-01A-12R-A414-31,TCGA-VQ-A8PU-01A-12R-A414-31,TCGA-HU-A4GY-01A-21R-A24K-31,TCGA-D7-6822-01A-11R-1884-13,TCGA-HU-A4GP-01A-11R-A251-31,TCGA-FP-7829-01A-11R-2055-13,TCGA-HU-8244-01A-11R-2343-13,TCGA-BR-8364-01A-11R-2343-13,TCGA-IN-A6RN-01A-12R-A33Y-31,TCGA-BR-4357-01A-01R-1157-13,TCGA-VQ-A8PE-01A-11R-A414-31,TCGA-VQ-AA6D-01A-11R-A414-31,TCGA-RD-A8MV-01A-11R-A36D-31,TCGA-ZA-A8F6-01A-23R-A36D-31,TCGA-RD-A8N5-01A-12R-A36D-31,TCGA-IN-A7NR-01A-11R-A354-31,TCGA-BR-8284-01A-11R-2343-13,TCGA-BR-A4J4-01A-12R-A251-31,TCGA-D7-6522-01A-11R-1802-13,TCGA-CG-4469-01A-01R-1157-13,TCGA-RD-A8N0-01A-12R-A36D-31,TCGA-BR-6565-01A-11R-1802-13,TCGA-IN-A6RL-01A-11R-A32D-31,TCGA-BR-8369-01A-11R-2343-13,TCGA-BR-7704-01A-11R-2055-13,TCGA-VQ-A8PF-01A-11R-A414-31,TCGA-BR-8679-01A-11R-2402-13,TCGA-VQ-AA6A-01A-11R-A414-31,TCGA-IN-7806-01A-11R-2055-13,TCGA-FP-7998-01A-11R-2203-13,TCGA-BR-8081-01A-11R-2343-13,TCGA-BR-8382-01A-11R-2402-13,TCGA-FP-7916-01A-11R-2203-13,TCGA-BR-A4J6-01A-11R-A251-31,TCGA-D7-A747-01A-22R-A33Y-31,TCGA-BR-A4IV-01A-31R-A251-31,TCGA-RD-A7BT-01A-11R-A33Y-31,TCGA-CG-4465-01A-01R-1157-13,TCGA-KB-A93H-01A-11R-A39E-31,TCGA-BR-8588-01A-11R-2402-13,TCGA-BR-6707-01A-11R-1884-13,TCGA-BR-4280-01A-01R-1131-13,TCGA-D7-6519-01A-11R-1802-13,TCGA-HU-A4GH-01A-11R-A24K-31,TCGA-BR-6454-01A-11R-1802-13,TCGA-SW-A7EA-01A-12R-A354-31,TCGA-HF-7133-01A-11R-2055-13,TCGA-D7-A748-01A-12R-A32D-31,TCGA-CG-5722-01A-21R-1602-13,TCGA-R5-A7O7-01A-11R-A33Y-31,TCGA-D7-8579-01A-11R-2343-13,TCGA-MX-A5UG-01A-21R-A31P-31,TCGA-VQ-A8PP-01A-21R-A414-31,TCGA-D7-6524-01A-11R-1802-13,TCGA-RD-A7C1-01A-11R-A32D-31,TCGA-BR-6457-01A-21R-1802-13,TCGA-VQ-AA69-01A-11R-A414-31,TCGA-D7-8574-01A-13R-2343-13,TCGA-VQ-A8E3-01A-11R-A39E-31,TCGA-VQ-AA6K-01A-11R-A414-31,TCGA-CG-4443-01A-01R-1157-13,TCGA-VQ-A91E-01A-31R-A414-31,TCGA-BR-6564-01A-12R-1884-13,TCGA-HU-A4GC-01A-12R-A251-31,TCGA-BR-8368-01A-11R-2343-13,TCGA-BR-4256-01A-01R-1131-13,TCGA-BR-8676-01A-11R-2402-13,TCGA-RD-A8N4-01A-21R-A36D-31,TCGA-CD-A489-01A-11R-A24K-31,TCGA-VQ-A8DT-01A-11R-A36D-31,TCGA-RD-A8MW-01A-11R-A36D-31,TCGA-HF-7131-01A-11R-2055-13,TCGA-BR-6803-01A-11R-1884-13,TCGA-BR-A44T-01A-32R-A24K-31,TCGA-BR-8373-01A-11R-2343-13,TCGA-BR-8059-01A-11R-2343-13,TCGA-FP-A4BF-01A-12R-A36D-31,TCGA-CG-5726-01A-11R-1602-13,TCGA-CD-8532-01A-11R-2343-13,TCGA-CG-5719-01A-11R-1602-13,TCGA-CG-4301-01A-01R-1157-13,TCGA-D7-A6EY-01A-21R-A31P-31,TCGA-SW-A7EB-01A-11R-A354-31,TCGA-HU-A4H0-01A-11R-A251-31,TCGA-VQ-AA68-01A-11R-A414-31,TCGA-BR-7722-01A-31R-2203-13,TCGA-FP-8210-01A-11R-2343-13,TCGA-BR-A44U-01A-11R-A36D-31,TCGA-BR-6802-01A-11R-1884-13,TCGA-CD-8527-01A-11R-2343-13,TCGA-IN-AB1X-01A-11R-A39E-31,TCGA-RD-A8NB-01A-12R-A39E-31,TCGA-BR-A4CR-01A-11R-A24K-31,TCGA-BR-8384-01A-21R-2402-13,TCGA-BR-A4QL-01A-31R-A251-31,TCGA-VQ-A92D-01A-11R-A414-31,TCGA-HU-A4GJ-01A-11R-A251-31,TCGA-VQ-AA6G-01A-11R-A414-31,TCGA-BR-8077-01A-11R-2343-13,TCGA-CD-A48C-01A-11R-A24K-31,TCGA-IN-7808-01A-11R-2203-13,TCGA-R5-A7ZE-01B-11R-A354-31,TCGA-VQ-A8PC-01A-11R-A39E-31,TCGA-CD-5804-01A-12R-2055-13,TCGA-RD-A7BS-01A-11R-A32D-31,TCGA-VQ-A91N-01A-11R-A414-31,TCGA-B7-5818-01A-11R-1602-13,TCGA-BR-4191-01A-02R-1131-13,TCGA-CG-5734-01A-11R-1602-13,TCGA-VQ-A94O-01A-11R-A414-31,TCGA-B7-A5TN-01A-21R-A31P-31,TCGA-VQ-A927-01A-12R-A414-31,TCGA-HU-A4GF-01A-11R-A24K-31,TCGA-FP-7735-01A-11R-2055-13,TCGA-VQ-A8E7-01B-11R-A414-31,TCGA-BR-8483-01A-31R-2402-13,TCGA-VQ-A922-01A-11R-A414-31,TCGA-IN-A6RO-01A-12R-A33Y-31,TCGA-BR-8295-01A-11R-2343-13,TCGA-D7-6525-01A-11R-1802-13,TCGA-FP-8211-01A-11R-2343-13,TCGA-VQ-A8DU-01A-11R-A36D-31,TCGA-FP-8631-01A-11R-2402-13,TCGA-VQ-A94U-01A-12R-A414-31,TCGA-BR-7707-01A-11R-2055-13,TCGA-CD-A487-01A-21R-A24K-31,TCGA-KB-A93G-01A-11R-A39E-31,TCGA-BR-7958-01A-21R-2343-13,TCGA-D7-6520-01A-11R-1802-13,TCGA-CG-4442-01A-01R-1157-13,TCGA-BR-6458-01A-11R-1802-13,TCGA-VQ-A8PB-01A-11R-A39E-31,TCGA-BR-4367-01A-01R-1157-13,TCGA-BR-8291-01A-11R-2343-13,TCGA-BR-4366-01A-01R-1157-13,TCGA-BR-8487-01A-11R-2402-13,TCGA-HU-8610-01A-22R-2402-13,TCGA-BR-8366-01A-11R-2343-13,TCGA-VQ-A8DZ-01A-11R-A36D-31,TCGA-BR-8380-01A-11R-2343-13,TCGA-BR-8289-01A-11R-2343-13,TCGA-VQ-A91Y-01A-11R-A414-31,TCGA-CD-8526-01A-11R-2343-13,TCGA-BR-8381-01A-11R-2402-13,TCGA-HU-A4H2-01A-11R-A251-31,TCGA-CG-5718-01A-11R-1602-13,TCGA-VQ-A91U-01A-11R-A414-31,TCGA-D7-A6EV-01A-11R-A31P-31,TCGA-BR-6710-01A-11R-1884-13,TCGA-IN-A6RS-01A-12R-A354-31,TCGA-R5-A805-01A-11R-A36D-31,TCGA-BR-8058-01A-31R-2343-13,TCGA-CD-A486-01A-11R-A24K-31,TCGA-IN-A6RR-01A-12R-A32D-31,TCGA-HU-A4H6-01A-11R-A251-31,TCGA-CD-8534-01A-11R-2343-13,TCGA-BR-6705-01A-12R-1884-13,TCGA-VQ-A928-01A-11R-A414-31,TCGA-D7-A4Z0-01A-22R-A251-31,TCGA-D7-8573-01A-11R-2343-13,TCGA-CG-4306-01A-01R-1157-13,TCGA-F1-A72C-01A-21R-A33Y-31,TCGA-VQ-A8E0-01A-11R-A414-31,TCGA-HU-A4H3-01A-21R-A251-31,TCGA-CG-4460-01A-01R-1157-13,TCGA-CG-4436-01A-01R-1157-13,TCGA-CD-A48A-01A-12R-A36D-31,TCGA-HF-7132-01A-11R-2055-13,TCGA-CD-8533-01A-11R-2343-13,TCGA-D7-8575-01A-11R-2343-13,TCGA-KB-A93J-01A-11R-A39E-31,TCGA-CG-4437-01A-01R-1802-13,TCGA-HU-8238-01A-11R-2343-13 |
