# Supplementary material for: Blockade of ITGA2 Induces Apoptosis and Inhibits Cell Migration in Gastric Cancer
Source: Biol Proced Online. 2018 May 1;20:10. doi: 10.1186/s12575-018-0073-x (PMC5928594; doi:10.1186/s12575-018-0073-x)
Supplement: Supplementary file 2 — Table S1. Primer list for reverse transcription-polymerase chain reactions (RT-PCR). (DOCX 15 kb) [file 12575_2018_73_MOESM2_ESM.docx]

**Table S1.** Primer list for reverse transcription-polymerase chain reactions (RT-PCR).

| **Gene** | **Sequence** |
| --- | --- |
| ITGA2 | F: 5'-AACAAGCATTCCAAATGTTACTGA-3' |
|  | R: 5'-AACTACCATTACTTTCGTAGCACT-3' |
| GAPDH | F: 5'-CCAGCCGAGCCACATCGCTC-3' |
|  | R: 5'-ATGAGCCCCAGCCTTCTCCAT-3' |
| Ras | F: 5'-TCCCCATGACACAATCCAGCT-3' |
|  | R: 5'-TTCCAGTTGACTGCAGACGTGTAT-3' |
| RhoA | F: 5'-AGTCTTCAGCAAGGACCAGTTCC-3' |
|  | R: 5'-TGCCATATCTCTGCCTTCTTCAG-3' |
| Bax | F: 5'-ACCAAGAAGCTGAGCGAGTGTC-3' |
|  | R: 5'-AATGTCCAGCCCATGATGGTT-3' |
| p38 | F: 5'-TGAACAAGACAATCTGGGAGGTG-3' |
|  | R: 5'-TCAGATCTGCCCCCATGAGAT-3' |
| Bim | F: 5'-AGTTCTGAGTGTGACCGAGAAGGT-3' |
|  | R: 5'-TGTGAGCAGAAAAGCGGGTAA-3' |
| Bcl-2 | F: 5'-CCTGTGGATGACTGAGTACCTGAA-3' |
|  | R: 5'-CAGGCATGTTGACTTCACTTGTG-3' |
| Apaf-1 | F: 5'-TTGCTGCCCTTCTCCATGAT-3' |
|  | R: 5'-TCCCAACTGAAACCCAATGC-3' |
| Caspase-9 | F: 5'-GGAGGATTTGGTGATGTCGGT-3' |
|  | R: 5'-TGGGCAAACTAGATATGGCGTC-3' |
| LIMK | F: 5'-GGGGCATCATCAAGAGCA-3' |
|  | R: 5'-CAGCCAGTGTTCCAGCTT-3' |
| Rac 1 | F: 5'-CCCTATCCTATCCGCAAACA-3' |
|  | R: 5'-CGCACCTCAGGATACCACTT-3' |
| PAK | F: 5'-AAGACATCCAACAGCCAGAA-3' |
|  | R: 5'-TGTAGCCACGTCCCGAGT-3' |
| CDC42 | F: 5'-GTGCCTGAGATAACTCACCAC-3' |
|  | R: 5'-CCTTCCTGTGTAAGTGCAGAA-3' |
| N-WASP | F: 5'-CCCCCAAATGGTCCTAATCT-3' |
|  | R: 5'-ACATGTCCAATGTCTGGAA-3' |
