# Supplementary figures and images for: Blockade of ITGA2 Induces Apoptosis and Inhibits Cell Migration in Gastric Cancer
Source: Biol Proced Online. 2018 May 1;20:10. doi: 10.1186/s12575-018-0073-x (PMC5928594; doi:10.1186/s12575-018-0073-x)

## Slide 1
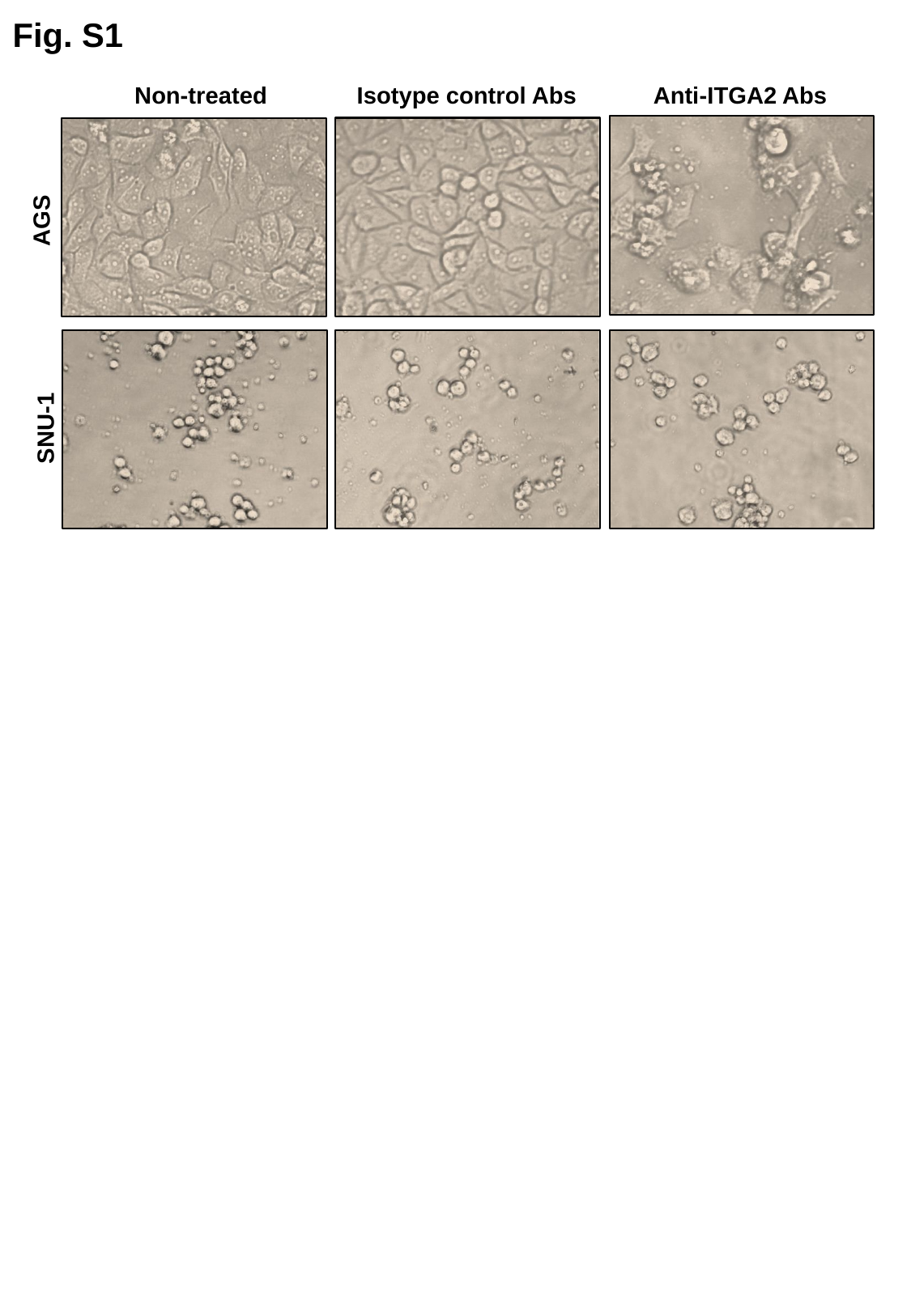

Fig. S1
Non-treated
Isotype control Abs
Anti-ITGA2 Abs
AGS
SNU-1

Supplement: Supplementary file 3 — Figure S1. Effect of anti-ITGA2 antibody on cell morphology. The AGS cells were treated with a 3 μg of the anti-ITGA2 antibodies or isotype control antibodies (negative control) for 48 h, and cell morphology was observed at 200X magnification. Data are representative of three independent experiments. (PPTX 1463 kb) [file 12575_2018_73_MOESM3_ESM.pptx]

## Slide 1
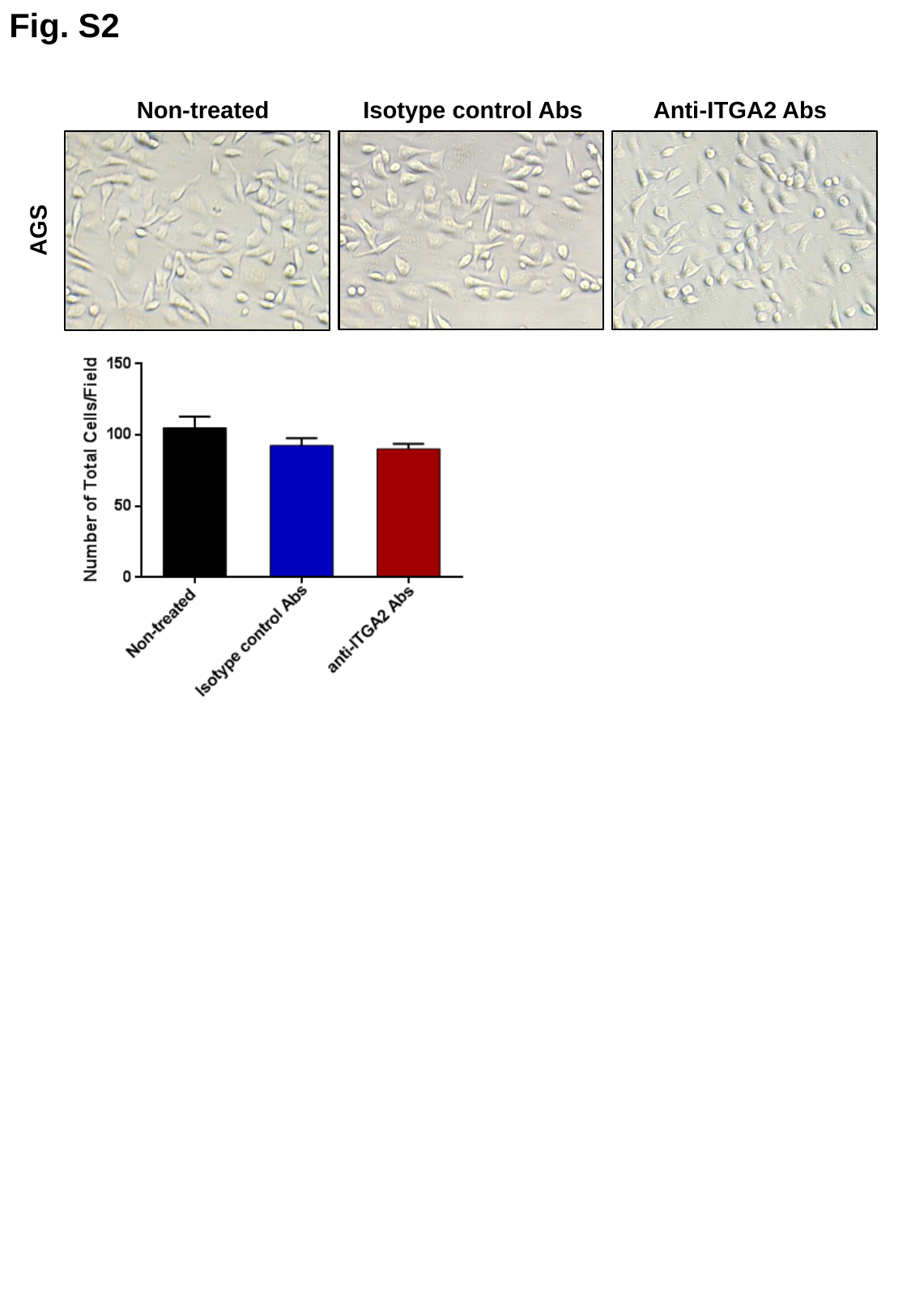

Fig. S2
Non-treated
Isotype control Abs
Anti-ITGA2 Abs
AGS

Supplement: Supplementary file 4 — Figure S2. Low dose of anti-ITGA2 antibodies did not induce cell death in AGS cells. Photography and quantitative analyses on cell number of the AGS cells treated with 0.1 μg anti-ITGA2 antibodies or isotype control antibodies (negative control) for 18 h. Data are expressed as mean ± standard deviation (S.D). Statistical comparisons were made by one-way ANOVA with Bonferroni comparisons. Data are representative of three independent experiments. (PPTX 784 kb) [file 12575_2018_73_MOESM4_ESM.pptx]
